# Tumor-Like Brain Lesions Associated With Variants of Uncertain Significance Compared to Previous Studies: A Case Report

**DOI:** 10.7759/cureus.26844

**Published:** 2022-07-14

**Authors:** José Omar Santellán-Hernández, Gerardo Romero-Luna, Jacqueline Ramírez-Cruz, Keren Magaly Aguilar-Hidalgo, Sonia Iliana Mejía-Pérez

**Affiliations:** 1 Neurosurgical Oncology Department, Instituto Nacional de Neurología y Neurocirugía "Manuel Velasco Suárez", Mexico City, MEX; 2 Radio-Neurosurgery Department, Instituto Nacional de Neurología y Neurocirugía "Manuel Velasco Suárez", Mexico City, MEX

**Keywords:** mri spectroscopy, cranial magnetic resonance imaging, vcan, pd6a, pseudotumor cererbi, cerebral leukodystrophy, trex1

## Abstract

TREX1 is a gene that encodes an exonuclease on the C-terminal strand at the 3 ́ end for DNA repair. Multiple syndromes associated with the alteration of this gene have been described, focusing in this case on retinal vasculopathy with cerebral leukodystrophy (RVCL). We present the case of a 44-year-old female patient with a familial history of cerebral pseudotumors. At the time of diagnosis, the patient presented weakness in the lower limbs and dysesthesias of the right body at the beginning of the clinical picture, without visual alterations or retinal changes at fundus examination. A cranial magnetic resonance imaging (MRI) study showed a pseudotumoral lesion at the inferior frontal gyrus with a report of a choline peak in spectroscopy, ring enhancement in contrasted T1 sequence, and apparent central necrosis. A molecular study shows a mutation in c2136G>A, c.799dup, and c.5312A>G related to genes expressing PDE6A, TREX1, and VCAN proteins, respectively, mutations that have not been previously reported.

## Introduction

Retinal vasculopathy with cerebral leukodystrophy (RVCL) was first reported in 1988 in a family with a familial history of unusual brain tumors [1]. RVCL is now known to be an autosomal dominant adult-onset small vessel disease caused by mutations that result in the translocation of the DNA exonuclease TREX1 [2,3].

Patients with RVCL exhibit a central constellation of neurological and visual symptoms [4]. Clinically, the disease presents in patients aged 35 to 40 years and is characterized by vascular retinopathy, Raynaud's phenomenon, migraine, and multiple internal organ dysfunction including renal disease, liver disease, gastrointestinal bleeding, anemia, and subclinical hypothyroidism [5,6].

In the most severe cases, patients present with vision loss, seizures, hemiparesis, apraxia, dysarthria, or memory loss. There can be progression to blindness, a neurovegetative state and death within 5 to 10 years after the onset of symptoms [6]. In this article, we present a clinical picture of cerebral leukodystrophy associated with a new variant of TREX1 never described before.

## Case presentation

We present the case of a 44-year-old Mexican female, widow, high school graduate, housewife, and right-handed, with a family history of a mother with an unspecified brain tumor, as well as a maternal grandmother and cousin diagnosed with retinal vasculopathy with cerebral leukodystrophy due to TREX1 mutation. She smoked two cigarettes per day for the past 28 years while denying other drug addictions. Blood type O positive. History of systemic arterial hypertension diagnosed in 2020 in control and trigeminal neuralgia diagnosed in 2017 in treatment with pregabalin. Allergy to ketorolac and carbamazepine.

She began in February 2021, five months prior to her first consultation, with weakness in the pelvic limb and right arm that made it difficult to ambulate; she was treated with analgesics and rehabilitation without improvement, which is why she requested medical evaluation.

In her initial evaluation, she presented with hemiparesis and dysesthesia of the right body, predominantly in the distal lower limbs. The Ashworth scale scored 1 spasticity in the right arm and the Ashworth scale scored 2 in the right leg. Hoffman and Tromner's signs are present on the right body.

**Figure 1:**
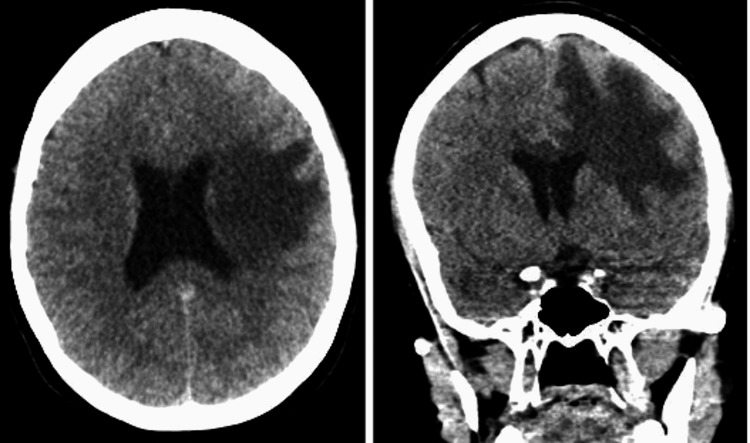
Simple cranial CT scan February 07, 2021 Axial (left) and coronal (right) sections, periventricular edema is observed in the left frontal region, with digitiform characteristics without displacement of the midline structures, without occupational lesion.

The patient had a simple cranial computed tomography (CT) scan performed externally with left frontal periventricular digitiform edema (Figure 1). A dense no proportionate complete right pyramidal syndrome and suspicion of a malignant brain tumor was integrated, so we administered prednisone 25 mg orally every 24 hours for management of brain edema due to tumor versus pseudotumoral lesion due to a family history of TREX1 mutation.

**Figure 2:**
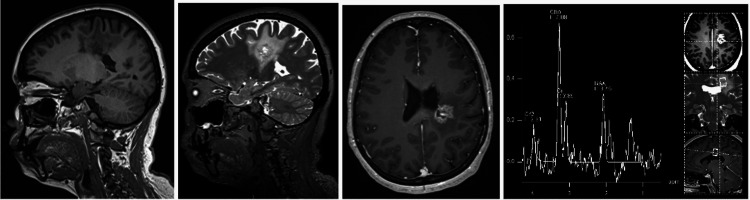
Cranial MRI April 13, 2021 From left to right: T1-weighted sagittal section, T2-weighted sagittal section, contrasted axial section, spectroscopy. There is a lesion in superior frontal gyrus, subcortical and periventricular, 17 mm in diameter, with neoplastic characteristics, hypointense in T1, hyperintense in T2 and ring enhancement with contrast and apparent central necrosis, in addition to presenting digitiform edema around the lesion.

Two months later, an oppressive biparietal headache of 4/10 was added. A contrasted cranial magnetic resonance imaging (MRI) was performed (Figure 2), showing a possible neoplastic lesion in the superior frontal gyrus with apparent central necrosis and a choline peak in the spectroscopy. Oncological neurosurgery service (NcxO) decided to admit the patient to the surgical programming list for a biopsy of the lesion; meanwhile, she was kept under medical management with steroids and was sent to the genetics service for evaluation and diagnostic approach due to the aforementioned family history. It was decided to perform a molecular study that reported alterations in the TREX1, VCAN, and PDE6A genes (Table 1) as variants of uncertain significance (VUS), which suggests a variant of retinal vasculopathy with cerebral leukodystrophy due to left frontal pseudotumoral lesion caused by TREX1. Management with steroids, physical rehabilitation sessions, and follow-up with neuro-oncology, genetics, and internal medicine services are maintained.

**Table 1:** Patient´s genetic mutations and their possible related diseases

Gene	Mutation	Related diseases
PDE6A	c2136G>A (p.Met.712Ile)	Retinitis pigmentosa (AR) - periventricular nodular heterotopia (AD)
TREX1	c.799dup (p.Ser267Lysfs*58)	Aicardi-Goutieres syndrome 1 (AR) - Chilblain lupus (AD) - retinal vasculopathy with cerebral leukodystrophy (AD) - systemic lupus erythematosus susceptibility
VCAN	c.5312A>G (p.LYS1771Arg)	Wagner syndrome and retinitis pigmentosa (AD) - related to congenital heart defects and early teeth loss

Four months later, in a follow-up visit, the patient presented with gait impossibility and difficulty in articulating words, in addition to a decrease in bilateral visual acuity, so an assessment was performed by the neuro-ophthalmology service (NO), which did not find any pathological alterations. An adjustment in steroid dose was made, achieving improvement in the symptoms. The histopathological study was deferred by NcxO since there was no benefit in performing the biopsy based on literature reports.

**Figure 3:**
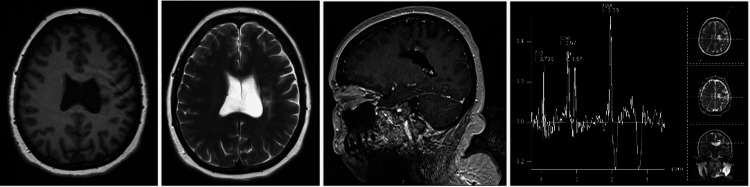
Cranial MRI February 05, 2022 From left to right: axial section in T1 weighting, axial section in T2 weighting, contrasted sagittal section, spectroscopy. The pseudotumoral lesion persists in the topography of the superior and middle frontal gyri, precentral gyrus, semioval center, corona radiata, the most caudal portion of the lenticular nucleus, posterior arm of the internal capsule, part of the optic radiations and the cerebral peduncle on the left side, as well as the corpus callosum. The lesion is hypointense in T1 and hyperintense in T2; decreased contrast ring enhancement; decrease of the choline peak and increase of the NAA peak.

In May 2022, a control MRI study was performed (Figure 3), where a reduction in the size of the lesion of approximately 50% was observed, which is attributed to the steroid treatment. Clinically, she continues with right-body hemiparesis.

## Discussion

The TREX1 gene, located on chromosome 3p21.31, encodes a 314 amino acid polypeptide with a molecular weight of ~33 kDa. Homologs of TREX1 are found in metazoans, are well conserved, and are expressed in most tissues, likely indicating an important maintenance function in most cells [2].

Mutation of this gene has been consistently linked to a wide range of acronyms for several similar pathologic phenotypes (Table 2) [3]. The cause of vascular retinopathy with cerebral leukodystrophy is mainly associated with a C-terminal frameshift mutation [5,6]. It is therefore a poorly known genetic disease. To date, just over 200 individuals worldwide have been reported with this condition [7,8].

**Table 2:** Diseases related to TREX1 gene mutation

Disease	Acronym
Retinal vasculopathy with cerebral leukodystrophy	RVCL
Cerebral hereditary angiopathy with vascular retinopathy and impaired other organs caused by TREX1 mutations	CHARIOT
Hereditary endoteliopathy with retinopathy, nephropathy and stroke	HERNS
Cerebrorretinal vasculopathy	CRV
Hereditary vascular retinopathy	HVR

In general, patients begin with vision problems, commonly reporting blurred vision or neurological symptoms such as paresthesias or plegia of a limb or hemibody. This disease becomes fatal 5-10 years after diagnosis [9,10].

The imaging study of choice is MRI, where a tumor-like lesion is revealed in about half of the patients, resembling a primary malignant neoplasm of the brain [9], while in the other half there are multiple small lesions in the white matter that may be misdiagnosed as demyelinating disease, brain abscess, immune reconstitution syndrome in AIDS, Sjögren's syndrome, and systemic lupus erythematosus (SLE) [11,12].

Previously, three types of lesions have been described on MRI; (1) white matter lesions (WML) without focal or confluent enhancement; (2) WML with punctate enhancement; and (3) lesions with rim enhancement with surrounding T2 hyperintensity (edema/gliosis) and/or diffusion restriction [12,13]. These lesions with surrounding edema may enlarge, causing a mass effect, and are called pseudotumors [14]. There are several infections that have been associated with vasculitis and can affect the central nervous system, including tuberculosis, syphilis, herpes simplex, and fungal and bacterial meningitis.

The treatment of these tumor-like or pseudotumoral lesions is not clear. This situation may lead to the erroneous behavior of surgical intervention, even if a biopsy is contraindicated since only a histopathological report of inflammatory cells would be found [14]. Steroids have not proven to be useful for the inflammatory component. Although the symptoms may stabilize, the prognosis of this condition is generally poor due to its progressive nature [14]. Moreover, the authors have seen no benefit with plasmapheresis, IV immunoglobulin, or chronic immunosuppression [15]. The efficacy of antiproliferative or biologic agents such as cyclophosphamide, azathioprine, and natalizumab is controversial. Future studies should focus on therapeutic agents such as tofacitinib and hydroxychloroquine because they reduce intracellular levels of interferon-α and β-interferon [15].

The present case is one of the few published reports of cerebral leukodystrophy without retinal damage. Our experience highlights the need to consider this pathology as a differential diagnosis in patients with tumefactive brain lesions, a family history of cerebral pseudotumors, and progressive visual involvement, which may help to avoid unnecessary invasive examinations. Likewise, contrary to the literature, the patient did not show clinical improvement with the use of steroids, but a decrease in the size of the pseudotumoral lesion was observed in MRI, probably due to the administration of steroids [16].

## Conclusions

Since previous findings and the results of this study show a clinical and imaging improvement just under oral steroid treatment, molecular testing for genetic mutations should be suggested in cases of vascular retinopathy, focal neurological deficits, and ring-enhancing lesions by long-standing imaging studies, as well as a family history of cerebral pseudotumors.

Trex1 mutation is not completely studied and since we have discovered more cases with this mutation at our institution, it should be a crucial area of research for geneticists, neurosurgeons, and neurologists. Surgical operations that are unnecessary for the patient's health and would not make any difference in the outcome. The Medical treatment for this disease should be studied as well. Our patient was treated with steroids, but only to treat the neurological symptoms she was experiencing. However, as we have seen in other cases that have been reported in the literature, the cerebral pseudotumor recurs and worsens over time, eventually leading to the patient developing irreversible neurological changes. 
